# Herbicide use and weed management strategies in hemp cultivation

**DOI:** 10.1186/s42238-025-00280-0

**Published:** 2025-05-16

**Authors:** Navneet Kaur, Anil Kumar, Tabarak Malik, Madhuri Girdhar, Manbir Singh, Reenu Singh, Mohd. Tariq, Anand Mohan

**Affiliations:** 1 School of Bioengineering and Biosciences, Lovely Professional University, Phagwara, Punjab India; 2 Gene Regulation Laboratory, National Institute of Immunology, New Delhi, India; 3 Department of Biomedical Sciences, Institute of Health, Jimma University, Jimma, Ethiopia; 4 Division of Research and Development, Lovely Professional University, Phagwara, Punjab, India; 5 Chaudhary Devi Lal University, Sirsa, Haryana India; 6 Department of Life Sciences, Parul Institute of Applied Sciences, Parul University, Vadodara, Gujarat-391760 India; 7 Department of Biotechnology, Graphic Era (Deemed to Be University), Dehradun, Uttarakhand-248002 India

**Keywords:** *Cannabis sativa*, Industrial hemp, Hemp cultivation, Herbicide tolerance, Integrated weed management, Sustainable agriculture

## Abstract

Industrial hemp has experienced a resurgence in global cultivation due to its diverse applications in textiles, food, bioplastics, biofuels, and environmental benefits such as phytoremediation and carbon sequestration. However, optimizing hemp production remains challenging, particularly in weed management, where limited approved herbicides and varying regional weed pressures pose significant obstacles. Weed dynamics across different regions highlight the prevalence of problematic species like *Chenopodium album* and *Amaranthus* spp. While hemp’s rapid canopy closure and high planting densities can reduce herbicide dependence, early-season weed competition can significantly impact crop establishment and yield. This review explores the current state of weed management in hemp cultivation, highlighting cultural, mechanical, and chemical strategies. Additionally, it evaluates the efficacy and phytotoxicity of pre- and post-emergent herbicides. Recent trials indicate that pre-emergent pendimethalin is consistently safe across multiple studies, while post-emergent grass herbicides like quizalofop, clethodim, and fluazifop provide effective control with minimal crop injury. For broadleaf control, clopyralid and bromoxynil show relative safety, though varietal responses vary. The limited availability of registered herbicides underscores the need for continued research and regulatory advancements. The review identifies critical knowledge gaps, including limited understanding of variety-specific herbicide tolerance and regional weed dynamics. Current research priorities include systematic herbicide screening across varieties and regions, optimizing cultural practices, and developing herbicide-tolerant cultivars. By integrating these strategies, hemp can fulfil its potential as a sustainable and profitable crop, contributing to environmentally friendly agricultural systems. This review provides a foundation for future research and policy decisions to optimize weed management in hemp production.

## Introduction

Industrial hemp (*Cannabis sativa* L.) has garnered renewed global interest due to its exceptional versatility, environmental benefits, and potential to contribute to sustainable agriculture (Kaur & Kander 2023). Historically cultivated for fiber, seeds, and medicinal purposes (Amaducci et al. 2015), hemp’s role in modern industries has expanded dramatically (Crini et al. 2020), driven by its applications in textiles (Mariz et al. 2024), bioplastics (Beluns et al. 2023), biofuels (Parvez et al. 2021; Tulaphol et al. 2021), and phytoremediation (Linger et al. 2002).

The renewed interest in hemp cultivation stems from its environmental and financial benefits, as well as legislative changes (Crini et al. 2020) that have reintroduced the crop to many countries' economies. Hemp is particularly notable for its environmental advantages. It requires significantly less water compared to cotton—up to 2.5 times less per unit area (Yano & Fu 2023)—and has lower agricultural input costs. Hemp also plays an important role in carbon sequestration and soil remediation, absorbing heavy metals (Linger et al. 2002) and improving soil health. Its ability to adapt to various climates and soil types makes it a promising option for sustainable agricultural systems (Enarevba & Haapala 2024; Tedeschi et al. 2022; Tripathi & Kumar 2022). These characteristics make hemp an attractive crop in the context of increasing demands for eco-friendly and resource-efficient solutions in agriculture and industry. Legislative changes, such as the 2018 U.S. Farm Bill and evolving policies in the European Union, have allowed hemp cultivation to expand in previously restricted regions (Duque Schumacher et al. 2020; Gitsopoulos et al. 2024). These changes have revitalized hemp industries globally, with Europe increasing its cultivated area from 20,540 hectares in 2015 to over 33,020 hectares by 2022 (European Commission 2024; Gitsopoulos et al. 2024).

Despite its potential, hemp cultivation faces several challenges that need to be addressed to optimize its production. Among these, weed management is a critical issue (Sandler & Gibson 2019). Unlike traditional crops, hemp cultivation lacks an established framework for effective weed control (Sandler & Gibson 2019). Weed competition is particularly problematic in the early stages of hemp growth (Gage et al. 2024), where its natural ability to suppress weeds through canopy closure is not yet fully established (Mettler 2021). If unmanaged, weeds can significantly reduce crop germination, plant density, and yield. While hemp is known for its competitive growth once established (Gitsopoulos et al. 2024), the early weed pressure can undermine its advantages (Pintilie et al. 2019), making effective management strategies essential (Schluttenhofer & Yuan 2017).

**Fig. 1:**
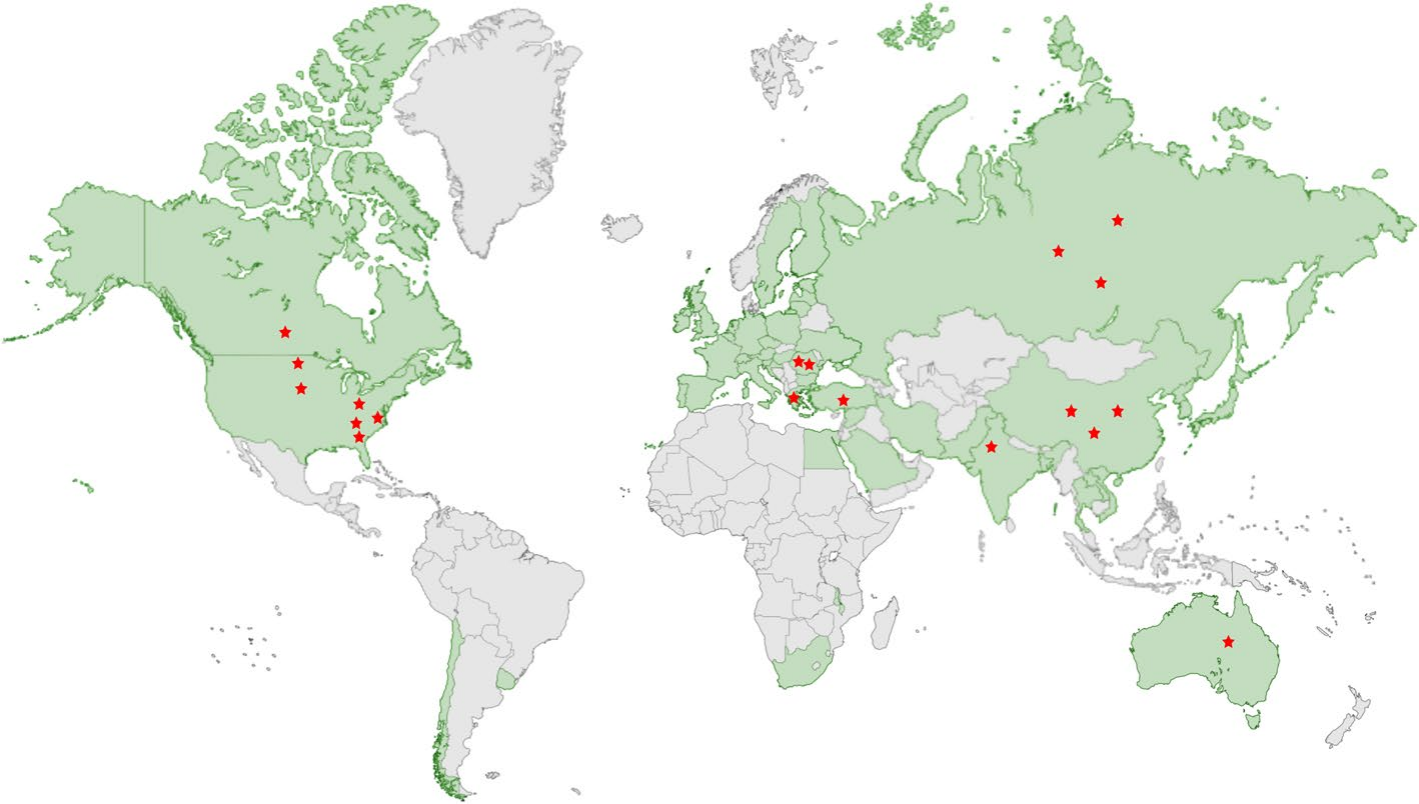
Hemp cultivation (Dhondt & Muthu 2021) and ongoing research in different countries and regions. Green denotes countries involved in hemp cultivation and red stars denote research involving herbicide application on hemp

One of the major barriers to weed management in hemp cultivation is the scarcity of approved herbicides (Ortmeier-Clarke et al. 2022; Singh et al. 2024). Globally, only a limited number of herbicides have been approved for use in hemp, in countries like Canada (Dupont 2016; Gowan 2018) and China (Liu et al. 2010, 2005; Song 2012) with USA (USEPA 2023) closely following behind. However, even where herbicides are available, the different hemp variety’s sensitivity to chemical inputs presents challenges. With the availability of large amount of commercially available herbicides, it is difficult to test each one for usage in hemp. A study used in silico methods for identifying target herbicides which might be tolerant to hemp (Kaur et al. 2024). This can narrow down the targets and expedite the trials for hemp cultivation. There is scarcity of research considering the large area under hemp cultivation as shown in Fig. 1. Therefore, research is needed to better understand how different herbicides affect hemp growth and yield, particularly in regions with diverse hemp varieties, weed pressures and environmental conditions (Sandler & Gibson 2019).

Weed management in hemp is not only a matter of improving crop yield but also a key aspect of sustainable farming practices. Hemp’s ability to suppress weeds naturally through its rapid growth, high planting densities, and biomass production offers an opportunity to reduce herbicide use. However, to fully harness these benefits, agronomic practices such as optimizing planting densities, row spacing, and cultivar selection must be tailored to specific growing conditions.

This review aims to provide a comprehensive overview of weed management strategies in hemp cultivation. It examines current practices, challenges, and emerging solutions, including cultural, mechanical, and chemical methods. This paper provides a foundation for advancing the economic and environmental benefits of hemp cultivation by fostering an understanding of weed dynamics, hemp’s natural weed suppressive abilities and weed management strategies. By consolidating existing research and identifying key gaps, this review aims to provide a foundation for informed decision-making and to guide future research priorities. Ultimately, advancing weed management in hemp cultivation will be pivotal to realizing its full potential as a sustainable and profitable crop in the global agricultural landscape.

## Weed dynamics in hemp cultivation across different regions

Industrial hemp cultivation is hindered by weed competition, which affects yield and quality. Studies reveal dynamic weed species composition influenced by environmental conditions, cultivation practices, and crop variety as shown in Table 1. Understanding these dynamics is crucial for devising region-specific, effective weed management strategies.

**Table 1:** Types of weed species in hemp crop in different regions

**Dicotyledonous Weed Species**	**Monocotyledonous Weed Species**	**Country**	**Year**	**Reference**
*Thlaspi arvense* L., *Capsella bursa-pastoris*, *Galium aparine* L., *Polygonum convolvulus* L., *Amaranthus retroflexus* L., *Chenopodium album* L., *Brassica napus* L	*Avena fatua* L., *Triticum aestivum* L	Saskatchewan, Canada	2000–2002	(Vera et al. 2006)
*Chenopodium album, Amaranthus retroflexus, Raphanus raphanistrum, Cirsium arvense, Convolvulus arvensis, Sonchus arvensis, Sinapis arvensis, Galinsoga parviflora, Polygonum persicaria, Polygonum convolvulus*	*Echinochloa crus-galli, Setaria viridis*	Secuieni, Neamt County, Romania	2006, 2007	(Chiriţă 2008)
*Chenopodium album, Euphorbia heterophylla, Bidens pilosa, Emex australis, Sonchus oleraceus*	*Cynodon dactylon, Urochlea panicoides, Cenchrus echinatus, Paspalum dilatatum*	Queensland, Australia	2010	(Hall et al., 2014)
*Abutilon theophrasti, Amaranthus retroflexus, Atriplex patula, Brassica rapae, Cirsium arvense, Convolvulus arvensis, Galinsoga parviflora, Polygonum persicaria, Sonchus arvensis, Solanum nigrum, Taraxacum officinale*	*Echinochloa crus-galli, Setaria glauca*	Secuieni, Neamt County, Romania	2018–19	(Pintilie et al. 2019)
*Amaranthus* spp., *Chenopodium album, Polygonum* spp., *Datura stramonium, Ipomoea* spp., *Solanum carolinense, Sorghum halepense*	*Digitaria sanguinalis, Eleusine indica, Echinochloa crus-galli, Setaria* spp., *Cyperus* spp.	Virginia, USA	-	(Britt et al. 2020)
*Artemisa vulgaris, Geranium pusillum, Thlaspi arvense, Chenopodium album, Taraxacum officinale, Euphorbia helioscopia*		Bydgoszcz, Poland	2021–2022	(Ambroziak et al. 2023)
*Chenopodium album, Portulaca oleracea, Convolvulus arvensis, Galinsoga quadriradiata, Solanum* spp., *Amaranthus* spp., *Ambrosia artemisiifolia, Persicaria* spp., *Abutilon theophrasti, Sinapis arvensis*	*Panicum dichotomiflorum, Setaria* spp., *Digitaria sanguinalis, Elymus repens, Cyperus esculentus*	USA		(Grab et al. 2023)
*Portulaca oleracea, Amaranthus retroflexus, Tribulus terrestris, Chenopodium album*	*Echinochloa crus-galli*	Athens, Greece	2019–2020	(Kousta et al. 2023)
*Chenopodium album, Amaranthus retroflexus, Tribulus terrestris, Solanum nigrum, Portulaca oleracea, Convolvulus arvensis, Abutilon theophrasti, Rumex crispus, Sonchus oleraceus, Cynanhum laeve*	*Cyperus* spp., *Sorghum halepense, Cynodon dactylon*	Thessaloniki and Arta, Greece	2022–2023	(Gitsopoulos et al. 2024)
*Amaranthus* spp., *Convolvulus arvensis*	*Cyperus esculentus, Setaria viridis*	Virginia, USA	2020–2023	(Podder et al. 2024)

Certain weeds, such as *Fallopia convolvulus*, *Avena fatua*, *Brassica napus*, and *Ipomoea* spp., challenge hemp cultivation. *Ipomoea* spp. is especially problematic in hemp seed production due to its similar seed size, complicating separation (Ehrensing 1998). A Saskatchewan study highlighted significant year-to-year variations in weed dominance, with *Thlaspi arvense* fluctuating from 60% in 2000 to 12% in 2001 and rising to 52% in 2002. Other species, such as *Capsella bursa-pastoris* and *Galium aparine*, also showed notable increases, alongside weeds like *Polygonum convolvulus*, *Amaranthus retroflexus*, and *Chenopodium album*. Weed density varied by cultivar, with Fasamo plots consistently hosting more weeds than Finola, despite taller Fasamo plants (Vera et al. 2006).

Research from Secuieni, Neamt County, identified annual monocotyledons like *Setaria viridis* (18 plants/m^2^) and *Echinochloa crus-galli* (23 plants/m^2^) as dominant weeds. Dicots included *Raphanus raphanistrum* (6 plants/m^2^), *Amaranthus retroflexus* (7 plants/m^2^) and *Chenopodium album* (9 plants/m^2^). Perennial weeds like *Cirsium arvense* and *Convolvulus arvensis* were also present, alongside *Sinapis arvensis* and *Polygonum convolvulus* (Chiriţă 2008).

Australian trials identified nine weed species, with *Chenopodium album* and *Cynodon dactylon* as the most dominant, followed by *Urochloa panicoides* and others (Bhattarai & Midmore 2014). Lithuanian studies documented 31 weed species, with *C. album* dominating in 2010, shifting to *Veronica arvensis* in 2011, and a more diverse community in 2012; however, diversity declined to eight species by harvest, with *C. album* and *Polygonum aviculare* persisting (Jankauskienė et al. 2014). Other trials highlighted *Fallopia convolvulus*, *Atriplex patula*, and *Polygonum persicaria* as dominant, with lower densities for *Cirsium arvense* and *Echinochloa crus-galli* (Pintilie et al. 2019).

In North America, grassy weeds like *Digitaria sanguinalis*, *Eleusine indica*, and *Echinochloa crus-galli* and broadleaf weeds such as *Amaranthus* spp. and *Chenopodium album* are common in hemp fields, with perennial weeds like *Sorghum halepense* posing unique control challenges due to underground structures (Britt et al. 2020). A study showed *C. album* affected shorter Canadian cultivars, while taller French and Chinese cultivars suppressed weeds effectively (Clarke 2020). In Poland, *Artemisia vulgaris* and *Geranium pusillum* dominated fields, followed by *Thlaspi arvense*, *C. album*, and *Euphorbia helioscopia* (Ambroziak et al. 2023).

A study over two seasons identified 10 weed species, equally split between annuals like *Portulaca oleracea* and *Amaranthus retroflexus* and perennials like *Solanum elaeagnifolium* and *Convolvulus arvensis*, with perennials dominating the first season (78.6%) (Kousta et al. 2023). The second season saw increased densities of nitrophilous weeds despite herbicide use, underscoring the challenge of managing weeds across seasons and the need for adaptive strategies. Weed infestation significantly reduced seed yield components in hemp, with *Fedora 17* better suppressing weeds due to superior canopy closure compared to *Uso 31* (Kousta et al. 2023).

Researchers emphasized the need for effective weed control, particularly for *Chenopodium album* and *Sorghum halepense* in low-density hemp sowings. *C. album* dominated untreated fields at 38.5 plants/m^2^ in the Thessaloniki Experiment 2022, persisting until harvest due to its competitive ability. Herbicides in the Arta Experiment were less effective against perennial grasses like *Cynodon dactylon* and *S. halepense* (5.7 and 7.9 plants/m^2^, respectively), while other dicots such as *Amaranthus retroflexus* and *Convolvulus arvensis* were present at lower densities (Gitsopoulos et al. 2024). In 2020 and 2021 trials, pre-planting applications of Roundup (Glyphosate) controlled grass species but failed to mitigate significant pressure from *Cyperus esculentus*, *Amaranthus* spp., and *Convolvulus arvensis*. Delayed herbicide applications in 2022 and 2023 due to unfavorable weather led to increased competition from *Amaranthus* spp. and *Setaria viridis* (Podder et al. 2024).

The persistence of problematic weeds like *Chenopodium album*, *Amaranthus retroflexus*, and *Cirsium arvense* highlights the need for integrated weed management, combining cultural practices, herbicides, and mechanical control. Studies show that adapting control methods to local conditions and cultivars is crucial for improving hemp yields. Continued research is vital for developing sustainable and effective weed management strategies in hemp cultivation.

## Natural weed suppression capabilities by hemp

Hemp is an effective natural weed suppressant due to its rapid growth, high planting density, and competitive resource utilization. Its ability to suppress weeds offers a sustainable alternative to herbicide-dependent farming. Cultivating hemp demonstrates that effective weed control can be achieved without chemical herbicides through appropriate agronomic practices.

### Role of planting density in weed suppression

Planting density is a key factor in hemp's ability to suppress weeds, with higher densities improving its competitive advantage. Research indicates that sufficient density allows hemp to control weeds naturally, reducing the need for herbicides (van der Werf et al. 1995). However, this effect diminishes at very low densities (10–30 plants/m^2^) (Lotz et al. 1991; van der Werf et al. 1995). Regional seeding rate recommendations vary, with higher rates suggested for fiber production in eastern Canada and Europe (Ranalli 1999), and increased rates (40–50 kg/ha) for seed production in Saskatchewan, Canada. Organic production benefits from higher rates (60–80 kg/ha) and narrow row spacing (18 cm), improving weed control, yield, and reducing weed size (Vera et al. 2002, 2006).

Studies show that plant density directly reduces weed biomass. Increasing density from 100 to 200 plants/m^2^ reduced weed biomass from 23.2 g/m^2^ to 6.5 g/m^2^. At 300 and 400 plants/m^2^, weed biomass further decreased to 2.6 g/m^2^ and 1.5 g/m^2^, respectively (Hall et al., 2014), indicating a non-linear relationship between density and weed suppression. Sufficient plant density eliminates the need for herbicides (Prade 2011; Reeves 2013). Seed rates also influence crop weediness (Jankauskiene et al. 2015).

Optimizing seeding rates based on crop purpose is essential for both weed control and economic viability. For fiber production, higher seeding densities (60–80 kg/ha) effectively suppress weeds while maximizing fiber yield (Vera et al. 2002, 2006; Vera & Hanks 2004). For hemp grain production, lower seeding rates (~ 30 kg/ha) result in a plant density of 100–150 hemp plants/m^2^, which is optimal for seed yield (Bócsa & Karus 1998). In Wales, higher planting densities (150 vs. 300 plants/m^2^) increased fiber yield across all five tested varieties and resulted in better weed suppression (Bennett et al. 2006).

Tailoring seeding rates to specific purposes is crucial. Insufficient sowing reduces yield, product quality, and increases weed competition, while excessive density leads to self-thinning and growth limitations in later stages (Hall et al., 2014). Hemp variety, growing season, soil type, and agronomic practices also influence the optimal density.

### Competitive growth and resource utilization

Hemp’s rapid growth and efficient resource utilization make it highly competitive against weeds. Its vigorous growth following emergence enables it to overshadow and outcompete weeds for sunlight, water, and nutrients (Hall et al., 2014; Kousta et al. 2023; Lotz et al. 1991; Thompson et al. 1998). A study highlighted hemp’s adaptability to diverse climates and its ability to thrive in low-nitrogen soils, make hemp particularly suitable for low-input agricultural systems (Zatta et al. 2012). Fast growth and dense foliage provide hemp with a natural advantage over weeds (Poisa & Adamovics 2010; Rehman et al. 2013). Hemp’s competitive ability is also influenced by agronomic practices such as row spacing and seeding rates as discussed above. The adaptability of hemp to different soil types and climates enhances its weed suppression potential across diverse agricultural systems.

### Allelopathic effects of hemp

Allelopathy is a key mechanism through which hemp suppresses weeds. While research on the allelopathic activity of *Cannabis sativa* is limited, its field dominance is often attributed to its aggressiveness and potential allelopathic effects on neighboring plants (McPartland 1997; Poonsawat et al. 2024; Ranalli 1999; Srivastava & Das 1974; Stupnicka-Rodzynkiewicz 1970). Hemp has been shown to produce allelochemicals, including terpenoids and cannabinoids, that inhibit the growth of both monocot and dicot weed species (Pudełko et al. 2014), providing an additional means of weed suppression beyond resource competition.

A greenhouse study on hemp residue found that even small amounts applied to the soil surface significantly reduced and delayed the germination of waterhemp seeds. This suggests that hemp residue, particularly through techniques like chaff-lining (spreading crop residue on the soil surface), can be incorporated into agricultural practices as an eco-friendly alternative to herbicides (Shikanai 2021).

Further research on hemp's potential to suppress *Amaranthus tuberculatus* (waterhemp), a notoriously difficult weed, found that hemp effectively suppressed its growth without herbicide use. This suppression occurred without significant yield loss, demonstrating that hemp can be successfully cultivated without relying on herbicides under certain conditions (Shikanai & Gage 2022).

### Environmental and long-term benefits

Hemp’s natural weed-suppressing properties provide significant environmental benefits by reducing the need for herbicides, thereby minimizing chemical runoff and soil contamination. This supports organic and sustainable agricultural practices (Amaducci et al. 2015; Stickland 1995). Hemp’s rotational benefits are well-documented, with studies showing its effectiveness in controlling both annual and perennial weeds (Sipos et al. 2010; Struik et al. 2000). In Europe, hemp cultivation significantly reduced *Cyperus esculentus* populations in subsequent corn crops, outperforming traditional rotations with barley or rye (Lotz et al. 1991). In Canada, hemp has been used to control thistles and couch grass (Jankauskienė et al. 2014). Hemp’s weed suppression improves soil health and biodiversity, reducing herbicide reliance and enhancing weed management in subsequent crops (Robson et al. 2002; Struik et al. 2000; van der Werf et al. 1996). Eight decades of research in Italy confirm hemp’s adaptability to various climates, low nitrogen requirements, and positive rotational effects, making it suitable for low-input agricultural systems (Zatta et al. 2012).

## Weed management strategies

**Fig. 2:**
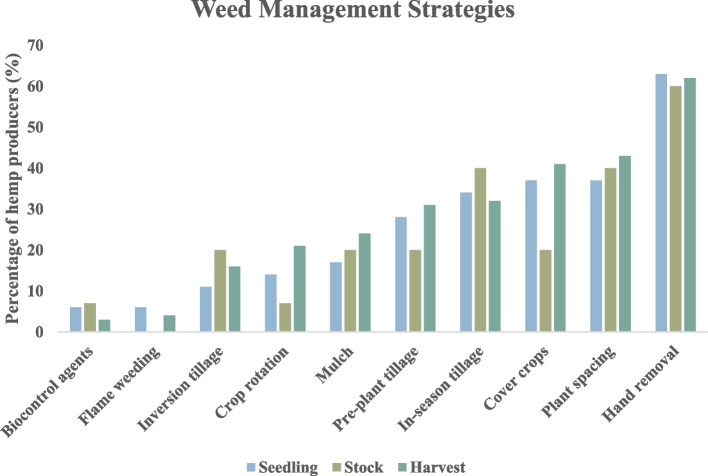
Percentage of hemp producers in various states of America who reported using various weed-control pest management methods for different end products – Seedling, Stock and Harvest in 2021 (Zavala et al. 2023). The terms “Seedling,” “Stock,” and “Harvest” follow the definitions from the original study, where “Seedling” refers to cultivation for seedlings, rooted cuttings, or tissue culture propagules, “Stock” represents mother or stock plants, and “Harvest” includes the final production of fiber, seeds, and inflorescence

Weed management is a significant challenge in global hemp production, with weeds reported as the most common pest, causing the highest yield losses and different farmers use various methods for weed management as shown in Fig. 2 (Zavala et al. 2023). Hemp’s natural competitiveness makes it a valuable tool in integrated weed management, helping reduce chemical input reliance while maintaining yields. However, hemp’s weed-suppressive capabilities are insufficient at low plant densities, particularly during early growth, which can lead to significant yield loss. Effective weed control is crucial when hemp is grown at low seeding rates, especially for seed or inflorescence production. Optimized planting densities and early planting are key to enhancing hemp's natural weed suppression (Kousta et al. 2023). Given hemp's slow initial growth and limited chemical control options, an integrated weed management approach is necessary.

### Cultural weed control

#### Crop rotation

**Fig. 3:**
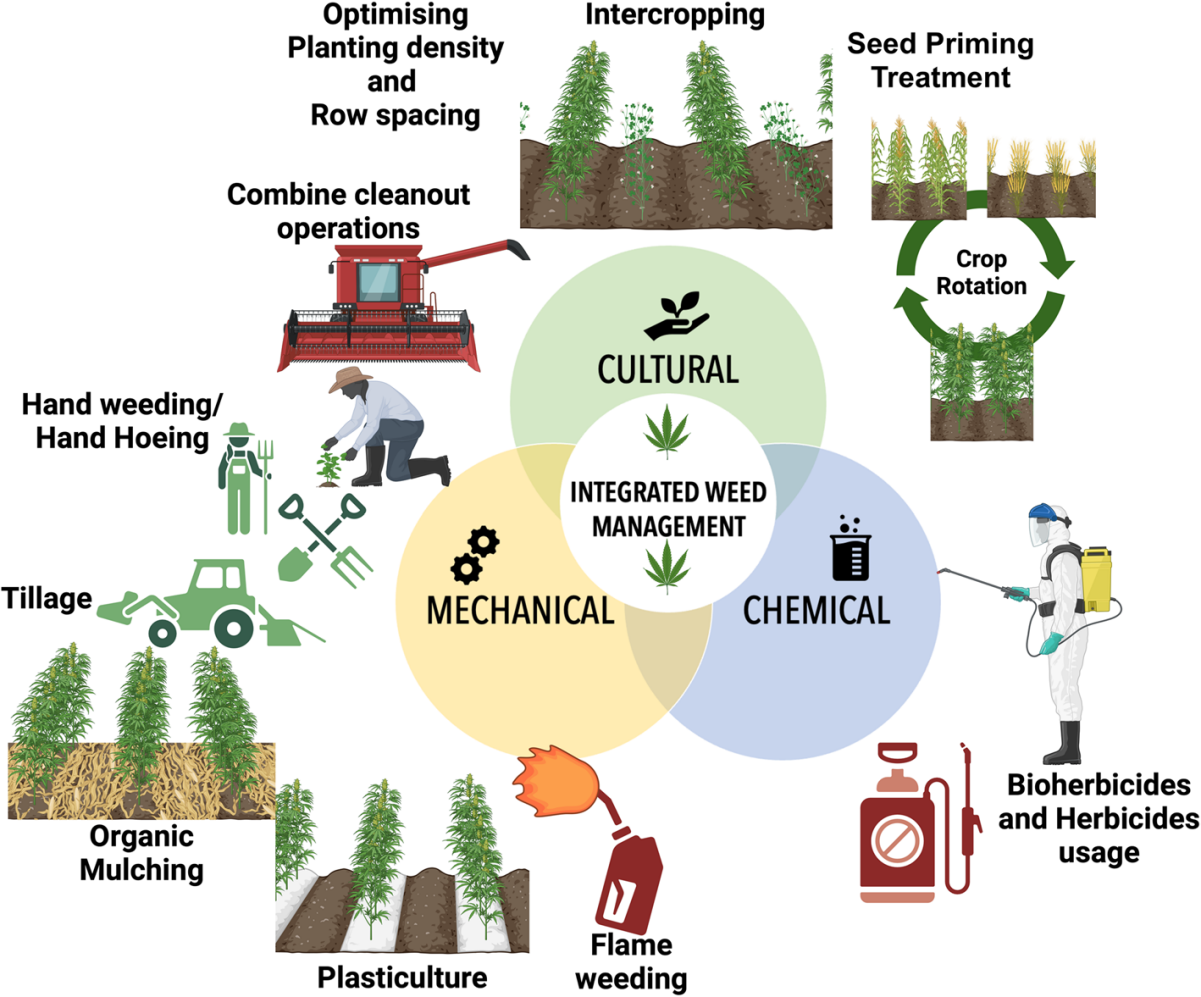
Weed management strategies in hemp cultivation

Crop rotation is a traditional and effective method for managing weed populations in hemp cultivation (Maxwell 2016; Sip et al. 2024; Velez Chavez 2023). A study emphasizes that rotating hemp with other crops helps reduce the buildup of weed species that are resistant to certain practices. Incorporating monocot crops like wheat (*Triticum aestivum* L.) and maize in a rotation with hemp will enhance the management of broadleaf weeds that could provide challenges in the subsequent year's hemp cultivation as shown in Fig. 3. Volunteer cash crops, including wheat, pea, canola and sunflower, can function as weeds and must be managed because of their competition with hemp plants for resources (Mettler 2021). Although crop rotation is not widely adopted among all hemp growers, a survey found that 7–21% of hemp growers employ this strategy, with varying success depending on the crop mix (Zavala et al. 2023).

#### Cover cropping

Cover cropping is frequently used as a preemptive strategy for managing weeds. The 2023 Cornell University study highlights rye or clover as effective cover crops for weed suppression as shown in Fig. 3 (Grab et al. 2023). These crops are often planted before the hemp and help reduce weed seed bank levels in the soil. The practice is also beneficial in terms of soil health, as cover crops improve soil structure and reduce erosion (Grab et al. 2023; Roth et al. 2020).Similarly, a survey indicates that 41% of harvest-stage hemp growers use cover cropping as part of their weed management toolkit (Zavala et al. 2023). Another study showed that cover cropping (cilantro, basil, marigold, sage, dill) significantly impacted the weed cover but had no effect on hemp yield and cannabinoid content. Therefore, cover cropping is a good strategy to maximize the benefits in hemp plantation (Connelly 2023).

#### Row spacing, plant density and seeding rate

Row spacing and increased seeding rates are crucial practices for managing weed pressure in hemp cultivation. Research shows that higher seeding rates (39 kg/ha) and narrower row spacing (7.6 cm) significantly reduce weed biomass compared to lower seeding rates (22 kg/ha) and wider spacing (40.6 cm) (Maxwell 2016). An Italian study further supports the importance of row spacing, demonstrating that hemp's competitive growth at higher seeding rates (40–120 plants/m^2^) helps suppress weeds by creating a dense canopy (Campiglia et al. 2017). Narrow row drilling for fiber crops is recommended to increase plant population and yield (Horner et al. 2019). Increased plant density improves weed suppression (Sunoj Valiaparambil Sebastian et al. 2023; Yazici 2023). However, excessive density may also lead to self-thinning in later stages, depending on variety-specific growth patterns (Bhattarai & Midmore 2014; van der Werf et al. 1995). Plant density can be adjusted depending on the desired end product, with denser sowing recommended for shorter fibers (Deng et al. 2019; Kumar et al. 2024). However, these practices are primarily suitable for fiber and grain hemp, which are typically grown at high densities to facilitate a dense canopy for weed suppression. In contrast, hemp cultivated for cannabidiol production requires a row spacing of 121–183 cm ensuring proper branching and airflow to minimize disease risk (Grab et al. 2023). This open canopy limits the effectiveness of density-based weed suppression, so alternative weed control methods such as mulching, cover crops, row cultivation, or mowing between rows are recommended due to lack of labeled herbicides (Grab et al. 2023).

#### Site selection and weed seed spread minimization

Strategic site selection is paramount in hemp cultivation. Fields with low weed densities and minimal perennial weed species are ideal. Preventative measures, such as harvesting clean fields first and thorough combine cleanout operations, help minimize the spread of weed seeds, ensuring long-term efficacy of weed management practices (Sosnoskie et al. 2024; Zavala et al. 2023).

#### Seed priming treatment

Hemp seeds are primarily cultivated for cannabinoid production, but their high cost and often low germination rates present a significant challenge (Shah et al. 2024). Optimizing germination is essential for improving crop establishment, reducing seed costs, and enhancing weed competitiveness. Seed priming is a pre-sowing treatment that conditions seeds to improve germination speed and uniformity (Farooq et al. 2019; Jisha et al. 2013). Various priming methods, including hydropriming, chemical priming, and nutrient-based priming, have been explored to enhance hemp seed performance. Among these, hydrogen peroxide (H_2_O_2_) priming (up to 1 M) and micronutrient priming with zinc (25 mM), selenium (12.5 mM), and manganese (12.5 mM) significantly improved germination by reducing oxidative stress and enhancing seedling vigor (Marks et al. 2022).

Experimental evaluations on multiple hemp cultivars, including Fedora-17, Finola, CBD Pink Kush, and Gulistan, showed that Indole Butyric Acid (IBA) priming (1000 ppm) significantly enhanced germination rates, with CBD Pink Kush achieving the highest response (83%). Hydropriming also promoted rapid seedling emergence. The highest seedling vigor index was observed in Fedora-17, CBD Pink Kush, and Gulistan following IBA treatment. A 24-h IBA (1000 ppm) priming or hydropriming with cocopeat as a growth medium is recommended for optimal hemp cultivation (Latif et al. 2025; Shah et al. 2024). Additionally, NaCl priming (250 mM, aerated, 4 days at 10 °C) accelerated early germination and reduced time to radicle protrusion, particularly in low-germinating seed lots (Tan et al. 2022). Solid matrix priming with Micro-Cel and osmotic priming with aerated KCl (1.15 MPa) improved early germination under supraoptimal temperatures but had limited impact on final germination percentage (Geneve et al. 2022). Gibberellin (GA3) pre-treatment improved germination, seedling growth, and drought tolerance by enhancing osmotic regulation and antioxidant enzyme activity. Optimal concentrations were 400 mg/L for ‘Yunma 1’ and 600 mg/L for ‘Bamahuoma’ (Du et al. 2022). Cold plasma treatment and iron (Fe) or manganese (Mn) nanoparticles (50 mg/L) improved germination and salt tolerance by enhancing chlorophyll content and reducing oxidative damage, with Fe nanoparticles and cold plasma (90 s) showing the best results (Ghasempour et al. 2024). Whereas magneto-priming showed no significant effect on germination (Spendier 2018).

While seed priming enhances germination and seedling vigor, ensuring optimal field conditions is crucial for translating these benefits into successful crop establishment (Sosnoskie et al. 2024). Proper seedbed preparation, including tillage to a depth of 30–40 cm in fall or winter, followed by a fine seedbed in spring, enhances seedling emergence (Campiglia et al. 2017; Desanlis et al. 2013). Optimal sowing depth (1–3 cm) ensures adequate moisture and seed-to-soil contact, promoting early canopy closure for weed suppression (Byrd 2019; Clarke 2020; Kousta et al. 2023; Mettler 2021). Shallower planting is preferred in moist soils, while a uniform depth of 3.8 cm is recommended for drier conditions; deeper seeding significantly reduces emergence (Roseberg et al. 2019).

### Mechanical weed control

#### Hand weeding

Hand weeding is a widely used method among hemp growers, with 60–63% employing it to manage weed pressure, especially during seedling, stock, and harvest phases (Zavala et al. 2023). While labor-intensive, it minimizes weed competition during critical growth stages (Kousta et al. 2023; Zavala et al. 2023). Hand weeding is particularly effective in systems with plastic mulch, where manual intervention is needed for weeds between rows (Britt et al. 2020). Smaller-scale operations, such as greenhouses, also rely on hand weeding due to its adaptability in controlled environments (Maxwell 2016). Early intervention through mechanical weed control is vital, as hemp’s slow initial growth makes it vulnerable to weed competition. Studies show that early hoeing at 15 and 30 days post-emergence significantly reduces weed density and prevents harmful competition (Campiglia et al. 2017). The hand-hoeing method has proven highly effective, improving crop growth by reducing nutrient, water, sunlight, and space competition, and boosting yield components in both growing seasons (Kousta et al. 2023).

#### Mechanical weeding

Tillage is commonly used in hemp production for weed control (Coolong et al. 2023; Roth et al. 2020; Velez Chavez 2023). Pre-plant ploughing helps prepare a seedbed and reduces the weed seed bank, minimizing competition for resources. Studies on organic hemp farmers in Canada highlight the effectiveness of post-emergence harrowing and pre-plant tillage in controlling weeds (Zavala et al. 2023). Secondary tillage, performed after planting, manages weeds between rows, especially in larger operations (Britt et al. 2020). The stale seedbed technique, which stimulates germination through tillage and sometimes irrigation, further reduces the weed seed bank by eliminating seedlings before planting, though it is time-intensive (Britt et al. 2020). A 2024 study outlined sequential tillage operations, including winter ploughing and harrowing, to ensure a clean seedbed before sowing (Sip et al. 2024). According to the 2023 Purdue survey, 31% of growers use pre-plant tillage to prepare the soil and control weeds, with in-season tillage more frequently used by stock growers (40%) (Zavala et al. 2023).

#### Mulching

Mulching is an effective weed control practice using both organic and synthetic materials as shown in Fig. 3. Organic mulches, such as straw, grass clippings, wood chips, and leaves, suppress weed growth by blocking light and preventing seedling emergence. They also improve soil health by regulating temperature, retaining moisture, and fostering beneficial microbial activity (Adesina et al. 2020). Plasticulture systems, commonly used in secondary metabolite hemp cultivation (Grab et al. 2023; Shikanai 2021; Wright 2022), combine plastic mulch with mechanical weed control methods. Plastic mulch significantly reduces weed emergence by blocking sunlight, and is often used in systems where hemp is grown for high-value products like cannabidiol (CBD) (Wright 2022). It helps modify soil temperature and moisture, providing an optimal environment for hemp growth while suppressing weeds (Shikanai 2021). However, managing weeds between rows remains a challenge, which can be addressed with mowing or low-growing ground covers (Britt et al. 2020; Shikanai 2021). Despite high input costs, the value of hemp makes plasticulture a preferred method. For instance, in Georgia, black plastic mulch combined with drip irrigation and herbicides has proven effective in reducing weed competition and optimizing hemp growth (Coolong et al. 2023).

#### Flame weeding

Flame weeding, a specialized technique, uses directed flames to damage weed tissues at the base of hemp plants. While highly effective, this method requires precision to avoid harming the crop. Straight rows and controlled flame applications are critical to minimizing the risk of crop damage during this process (Knezevic & Scott 2020; Zavala et al. 2023).

### Chemical weed control

Chemical control options for hemp cultivation are limited due to fewer studies on their effect on hemp plants. While some organic herbicides such as caprylic acid and ammonium nonanoate have been used, they require careful application to avoid damaging the hemp crop (Grab et al. 2023). In Canada, registered herbicides include pre-plant incorporated ethalfluralin (85–140 g ai ha^-1^), which is a seedling inhibitor and post-emergence quizalofop-p-ethyl (93 g ai ha^-1^), an acetyl-CoA carboxylase (ACCase) inhibitor herbicide for grass control (Mettler 2021). Notably, in April 2023, New York State approved a supplemental label for Sonalan® HFP (ethalfluralin), effective until May 2026 (USEPA 2023). This soil-applied herbicide, targeting annual grasses and select broadleaf weeds, requires application rates of 0.5625 to 1.125 lb ai/acre, with dosage adjustments based on soil characteristics (USEPA 2023).

European hemp cultivation generally employs minimal chemical intervention (Amaducci et al. 2015). Notable exceptions include the Czech Republic, where research has documented the use of linuron, a photosystem II inhibitor, for post-planting broadleaf weed control (Tang et al. 2016). In contrast, Italian production guidelines advocate for cultivation without herbicides, pesticides, or fertilizers (Amaducci 2005). Chinese hemp production protocols endorse the pre-emergence application of S-metolachlor, acetochlor, and pendimethalin (Amaducci et al. 2015).

## Evaluating herbicide options for effective weed management in hemp production

The growing demand for hemp as a versatile crop in industries ranging from textiles to biofuels necessitates a re-evaluation of traditional weed management strategies. Conventional methods, such as manual weeding and mechanical cultivation, are increasingly inadequate for large-scale production systems. These approaches are labor-intensive, time-consuming, and often fail to address the scale and intensity of weed infestations that compromise hemp yield and quality. Consequently, chemical weed control emerges as a critical solution to meet the challenges of modern hemp agriculture. Research highlights that under dry or unfavorable conditions, or when plant populations were low, weed infestations could pose a significant challenge to hemp cultivation (Vera & Hanks 2004). A pivotal study in Illinois and Virginia tested the impact of cultural and chemical weed control tactics on the yield and weed community in dual-purpose hemp production, particularly in light of limited herbicide options. The research, carried out in Illinois and Virginia, tested a 3 × 2 × 3 factorial design, including seeding rates (100, 200, or 300 plants m^-1^) and row spacings (19 or 38 cm), and herbicide treatments (non-treated, ethalfluralin followed by quizalofop, and S-metolachlor followed by clethodim). The results showed species richness was higher in Illinois, with nontreated plots at 19 cm row spacing and 100 plants m^-1^ exhibiting the highest richness. Herbicide treatments significantly reduced weed biomass, especially for waterhemp (*Amaranthus tuberculatus*) and broadleaf signalgrass (*Urochloa platyphylla*) in Illinois. Both herbicide programs resulted in lower weed biomass compared to the non-treated plots, but no significant difference was observed in Virginia. Hemp yield was higher in Virginia than in Illinois, and in Illinois, herbicide treatments increased yield, with S-metolachlor followed by clethodim showing the most significant impact. The study concluded that while cultural practices did not significantly affect weed biomass, herbicide programs were effective in reducing weed biomass and potentially increasing yield in regions with high weed pressure (Gage et al. 2024). Thus, this section reviews current research on pre- and post-emergence herbicide use in hemp cultivation as shown in Tables 2 and 3. It examines the potential phytotoxic effects of commercial herbicides on hemp growth, yield, and cannabinoid production.

**Table 2:** Phytotoxicity caused by various pre-emergent herbicides in different varieties of hemp across the world

75.1–100% injury	50.1–75% injury	25.1–50% injury	0–25% injury	Treatment Time	Observation Time	Variety	Experiment/City	Reference
		Metribuzin (0.375 kg ai/ha)	Acetaclor (1.98 kg ai/ha), Metolachlor (1.44 kg ai/ha)	4 days after planting	-	Zenit	Field/Romania	(Chiriţă 2008)
Mesotrione (0.39 L/ha)			Fomesafen (1.52 L/ha), Metolachlor (1.95 L/ha), Pendimethalin (2.80 L/ha), Pyroxasulfone (0.070 kg/ha),	on the day of planting	4 weeks after treatment	Italian Cultivars	Field/Kentucky, USA	(Maxwell 2016)
Fomesafen (1.52 L/ha), Mesotrione (0.39 L/ha), Metolachlor (1.95 L/ha)		Pendimethalin (2.80 L/ha), Pyroxasulfone (0.070 kg/ha)		on the day of planting	4 weeks after treatment	Finola	Field/Kentucky, USA	(Maxwell 2016)
	Clomazone (1.4 kg ai/ha), Norflurazon (2.8 kg ai/ha)	Acetochlor (3.4 kg ai/ha), Dimethanamid-P (0.7 kg ai/ha), Flumioxazin (0.1 kg ai/ha), Fomesafen (0.4 kg ai/ha), Metribuzin (0.6 kg ai/ha), Pyroxasulfone (0.9 kg ai/ha), S-metolachlor (1.6 kg ai/ha)	Chlorimuron (0.04 kg ai/ha), Diuron (2.3 kg ai/ha), Linuron (1.4 kg ai/ha), Pendimethalin (1.6 kg ai/ha)	on the day of planting	4 weeks after treatment	Felina 32	Greenhouse/Virginia, USA	(Byrd 2019)
Chlorimuron (0.04 kg ai/ha), Linuron (1.4 kg ai/ha)	Fomesafen (0.4 kg ai/ha)		Pendimethalin (1.6 kg ai/ha), S-metolachlor (1.6 kg ai/ha)	on the day of planting	30 days after treatment	Helena 2017	Field/Virginia, USA	(Byrd 2019)
		Chlorimuron (0.04 kg ai/ha), Pendimethalin (1.6 kg ai/ha)	Fomesafen (0.4 ai/ha), Linuron (1.4 kg ai/ha), S-metolachlor (1.6 kg ai/ha)	on the day of planting	30 days after treatment	Joey 2018	Field/Virginia, USA	(Byrd 2019)
Ethofumesate (3.361 kg ai/ha), Mesotrione (0.105 kg ai/ha), Metribuzin (0.277 kg ai/ha), Pyroxasulfone (0.109 kg ai/ha)	Acetochlor (1.053 kg ai/ha), Isoxaflutole (0.053 kg ai/ha)	Acetochlor (1.053 kg ai/ha), Atrazine (0.426 kg ai/ha), Dimethenamid-P (0.84 kg ai/ha), Saflufenacil (0.038 kg ai/ha), S-metolachlor (1.597 kg ai/ha), Sulfentrazone (0.158 kg ai/ha)	Imazethapyr-ammonium (0.044 kg ae/ha), Pendimethalin (1.12 kg ai/ha), Flumioxazin (0.089 kg ai/ha), Quinclorac-methyl (0.289 kg ai/ha), Trifluralin (0.56 kg ai/ha)	on the day of planting	25 days after emergence	CFX-2	Greenhouse/North Dakota, USA	(Mettler 2021)
			Acetochlor (1.05 kg ai/ha), Imazethapyr-ammonium (0.044 kg ae/ha), Pendimethalin (1.12 kg ai/ha), Pyroxasulfone (0.109 kg ai/ha), Quinclorac-methyl (0.29 kg ai/ha), Saflufenacil (0.038 kg ai/ha), Trifluralin (0.84 kg ai/ha),	on the day of planting	28 days after emergence	X-59	Field/North Dakota, USA	(Mettler 2021)
	Clomazone (0.3 kg, L/ha)	Metribuzin (1.2 kg, L/ha), S-metolachlor (1.5 kg, L/ha)	Aclonifen (4.0 kg, L/ha), Bifenox (1.5 kg, L/ha), Lenacil (0.8 kg, L/ha), Oxyfluorfen (0.5 kg, L/ha), Pendimethalin (3.0 kg, L/ha),	4 days after planting	4 weeks after treatment	Secuieni—Jubileu	Field/Romania	(Puiu et al. 2023)
	Clomazone (0.096 kg ai/ha)		Dimethenamid-P (0.576 kg ai/ha), Pendimethalin (1.65 kg ai/ha), Pyroxasulfone (0.1275 kg ai/ha)	on the day of planting	2 weeks after treatment	Finola	Pot/Yozgat, Turkey	(Kale et al. 2024)
			Clomazone (0.096 kg ai/ha), Dimethenamid-P (0.576 kg ai/ha), Pendimethalin (1.65 kg ai/ha), Pyroxasulfone (0.1275 kg ai/ha)	on the day of planting	-	Finola	Field/Yozgat, Turkey	(Kale et al. 2024)

**Table 3:** Phytotoxicity caused by various post-emergent herbicides in different varieties of hemp across the world

75.1–100% injury	50.1–75% injury	25.1–50% injury	0–25% injury	Treatment time	Observation time	Variety	Experiment/City	Reference
2,4-D, Ametryn, Amitrole, Atrazine, Bentazon, Diquat, Diuron, Ethofumesate, Fluometuron, Glyphosate, Karbutilate, Linuron, Methabenzthiazuron, Methazole, Metribuzin, Oxadiazon, Paraquat, Phenmedipham, Phenobenzuron, Prometryn, Terbutryn	Barban, Butralin, Dalapon, Difenzoquat, Dinitramine, Diphenamid, IPC, Penoxalin, Napropamide, Trifluralin, U-27267		Alachlor, Benefin, Benzthiazuron, Butylate, Chloroxuron, Cycloate, EPTC, Molinate, Nitralin, Nitrofen, Pebulate, Perfluidone, Pronamide, TFP, Vernolate	1–3 leaf pairs stage	-	USDA Plant introduction numbers 377927, 377,929, 377,935	Glasshouse/Israel	(Horowitz 1977)
Amitrole (2 kg/ha), Bentazon (2 ai kg/ha), Diquat + Praquat (0.4 ai kg/ha), Glyphosate (0.82 kg/ha), Ioxynil (1 kg/ha), Metribuzin (0.14 kg/ha), MSMA + cacodylate (1.9 ai kg/ha), Phenmedipham (0.66 ai kg/ha)		Bromoxynil (1 kg/ha)		10–20 cm tall	2 weeks after treatment	USDA-377927, 377,929, 377,935	Field/Israel	(Horowitz 1977)
Glyphosate (0.82 kg/ha)	Amitrole (2 kg/ha)	Ioxynil (1 kg/ha), Metribuzin (0.14 kg/ha)	Bromoxynil (1 kg/ha)	80 cm tall	2 weeks after treatment	USDA- 377,927, 377,929, 377,935	Field/Israel	(Horowitz 1977)
	Triasulfuron (0.03 kg ai/ha), Tribenuron methyl (0.01125 kg ai/ha)	Chlopyralid (0.09 kg ai/ha)	Fluosiphor-p-butyl (0.15 kg ai/ha)	2–3 leaf pairs stage	-	zenit	Field/Neamt,Romania	(Chiriţă 2008)
Bispyribac-Na (0.02 kg/ha), Flazasulfuron (0.11 kg/ha), Rimsulfuron (0.07 kg/ha), Trifloxysulfuron (0.007 kg/ha)			Bromoxynil (0.58 L/ha), MSMA 3.16 L/ha)	4–5 leaf pairs stage/30.5 cm tall	2 weeks after treatment	Italian cultivars	Field/Kentucky, USA	(Maxwell 2016)
	Trifloxysulfuron (0.007 kg/ha)	Flazasulfuron (0.11 kg/ha), Rimsulfuron (0.07 kg/ha)	Bispyribac-Na (0.02 kg/ha), Bromoxynil (0.58 L/ha), MSMA (3.16 L/ha)	4–5 leaf pairs stage/30.5 cm tall	2 weeks after treatment	Finola	Field/Kentucky, USA	(Maxwell 2016)
	Bentazon (5.6 kg ai/ha), Fomesafen (0.2 kg ai/ha), Halosulfuron (0.05 kg ai/ha), Imazaquin (0.8 kg ai/ha), Imazethapyr (0.2 kg ai/ha), Linuron (1.4 kg ai/ha), Thifensulfuron (0.02 kg ai/ha)	Acifluorfen (2.2 kg ai/ha), Bromoxynil (0.3 kg ai/ha), Chlorimuron (0.02 kg ai/ha), Clopyralid (0.1 kg ai/ha), Pyrithiobac (3.6 kg ai/ha), Quizalofop (1.0 kg ai/ha), Sethoxydim (0.3 kg ai/ha)		20–28 cm tall	4 weeks after treatment	Felina 32	Greenhouse/Virginia, USA	(Byrd 2019)
	Halosulfuron (0.05 kg ai/ha)		Bromoxynil (0.3 kg ai/ha), Clopyralid (0.1 kg ai/ha), Quizalofop (1 kg ai/ha), Sethoxydim (0.3 kg ai/ha)	20–28 cm tall	30 days after treatment	Helena 2017	Field/Virginia, USA	(Byrd 2019)
		Halosulfuron (0.05 kg ai/ha)	Bromoxynil (0.3 kg ai/ha), Clopyralid (0.1 kg ai/ha), Quizalofop (1 kg ai/ha), Sethoxydim (0.3 kg ai/ha),	20–28 cm tall	30 days after treatment	Joey 2018	Field/Virginia, USA	(Byrd 2019)
Flucarbazone-sodium (0.0157 g ai/ha), Fluroxypyr-methyl (0.14 kg ae/ha), Halauxifen-methyl (0.0053 kg ai/ha), Glyphosate-potassium (0.84 kg ae/ha), Glufosinate-ammonium (0.655 kg ai/ha), Paraquat (0.28 kg ai/ha), Tembotrione (0.092 kg ai/ha), Topramezone (0.0123 kg ae/ha)	Nicosulfuron (0.0256 kg ai/ha), Pyroxsulam (0.0151 kg ai/ha), Tribenuron-methyl (0.0088 kg ai/ha), 2,4-D Amine 4 (0.28 kg ai/ha), Metribuzin (0.21 kg ai/ha), Carfentrazone-ethyl (0.009 kg ai/ha), Fomesafen-sodium (0.198 kg ai/ha)	Cloransulam-methyl (0.0175 kg ai/ha), Halosulfuron-methyl (0.0352 kg ai/ha), Imazamox-ammonium (0.035 kg ai/ha), Imazethapyr-ammonium (0.035 kg ai/ha), Triflusulfuron-methyl (0.0315 kg ai/ha), Dicamba (0.07 kg ae/ha), Atrazine (0.28 kg ai/ha), Bentazon-sodium (0.28 kg ai/ha), Oxyfluorfen (1.1204 kg ai/ha)	Bromoxynil (0.28 kg ai/ha), Clopyralid (0.105 kg ae/ha)	3 leaf pairs stage/8–12 cm tall	19 days after treatment	CFX-2	Greenhouse/North Dakota, USA	(Mettler 2021)
Cloransulam-methyl (0.018 g ai/ha), Oxyfluorfen (1.12 kg ai/ha)		Atrazine (0.28 kg ai/ha), Bromoxynil (0.28 kg ae/ha), Imazamox-ammonium (0.035 kg ae/ha), Quinclorac-methyl (0.29 kg ai/ha)	Clopyralid (0.105 kg ae/ha)	3 leaf pairs stage/8–12 cm tall	21 days after treatment	X-59	Field/North Dakota, USA	(Mettler 2021)
Dicamba (0.560 kg ae/ha)	2,4-D (1.060 kg ae/ha)				28 days after treatment	Tangerine	Greenhouse/Ohio, USA	(Essman 2022; Essman et al. 2024)
Isoxaflutole (0.112 kg ai/ha)	Isoxaben (0.631 kg ai/ha), Halosulfuron (0.039 kg ai/ha)	Dithiopyr (0.28 kg ai/ha), Metribuzin (0.21 kg ai/ha), Prometryn (1.121 kg ai/ha), S-metolachlor (1.068 kg ai/ha)	Acetochlor (0.841 kg ai/ha), Flumioxazin (0.072 kg ai/ha), Fomesafen (0.21 kg ai/ha), Norflurazon (0.138 kg ai/ha), Oxyfluorfen (0.421 kg ai/ha), Pendimethalin (1.065 kg ai/ha)	1–2 leaf pairs stage	2 weeks after treatment	Von	Treated 1 day before pretransplant in field/Georgia, USA	(Wright 2022; Wright-Smith et al. 2024)
Halosulfuron (0.026 kg ai/ha), Metribuzin (0.21 kg ai/ha), Prometryn (1.121 kg ai/ha), Trifloxysulfuron (0.005 kg ai/ha)	Imazethapyr (0.035 kg ai/ha)		Acetochlor (0.841 kg ai/ha), Clethodim (0.136 kg ai/ha), Pendimethalin (1.065 kg ai/ha), S-metolachlor (1.068 kg ai/ha)	13–18 cm tall	2 weeks after treatment	Sunbelt Crush	Treated posttransplant in field/Georgia, USA	(Wright 2022; Wright-Smith et al. 2024)
	Bentazon (2.5 kg, L/ha)		Bromoxynil + 2,4-D(0.8 kg, L/ha), Clopyralid (0.5 kg, L/ha), Clethodim (1.5 kg, L/ha), Fluazifop-p-butyl (1.2 kg, L/ha)	25–30 cm tall/8–10 leaf pairs stage	4 weeks after treatment	Secuieni—Jubileu	Field/Botosani, Romania	(Puiu et al. 2023)
Bentazone (0.72 kg ai/ha)	Bromoxynil (0.3375 kg ai/ha)		Halosulfuron-methyl (0.030 kg ai/ha), Quizalop-p-ethyl (0.050 kg ai/ha)	30 days after planting		Finola	Field and Pot/Yozgat, Turkey	(Kale et al. 2024)
			Clethodim (0.272 kg ai/ha), Fenoxaprop (0.086 kg ai/ha), Fluazifop (0.210 kg ai/ha), Fluazifop + Fenoxaprop (0.21 + 0.059 kg ai/ha), Pinoxaden (0.06 kg ai/ha), Quizalofop (0.092 kg ai/ha), Sethoxydim (0.315 kg ai/ha)	20–25 cm tall/2–3 leaf pairs stage	21 days after treatment	NWG2730 and NWG452	Greenhouse/Nebraska, USA	(Zaric 2023)

### Pre-emergent herbicidal control

In a Romanian study, all the pre-emergent herbicides—acetachlor (1.980 kg ai/ha), metolachlor (1.44 kg ai/ha), and metribuzin (0.375 kg ai/ha) were tolerated by Zenit variety of hemp in field trials as shown in Table 2. These treatments resulted in an increase in seed yields from 225 kg/ha in untreated plots to 327–525 kg/ha with herbicide application. The most effective pre-emergent weed control was achieved with acetachlor (65%), metribuzin (60%), and metolachlor (55%), although metribuzin caused significant damage to the hemp plants (Chiriţă 2008). Amaducci et al. discussed weed management practices in China, where mechanical or chemical weed control is performed as needed. In Southern China, herbicidal weed control is prominent, where pre-emergent herbicides are applied to the soil after sowing. 30% pendimethalin EC (3 L/ha) or 65% metolachlor emulsion (3 L/ha) were recommended as effective herbicides in Heilongjiang Province (Song 2012). Researchers suggested using 96% metolachlor EC (1.05 L/ha) or 50% acetochlor EC (0.75 L/ha) for hemp fields, based on their studies in Hunan Province (Liu et al. 2010). Additionally, another group reported the use of 50% acetochlor EC (2.25 kg/ha) for weed control in Yunnan Province (Amaducci et al. 2015; Liu et al. 2005).

In the USA, research conducted experiments testing 5 pre-emergent herbicides at two different locations (Bowling Green and Lexington), using various cultivars (Italian cultivars and Finola), row spacings (7.6 cm and 40.6 cm), and seeding densities (39 kg/ha and 22 kg/ha). The results showed significant variation between the locations due to these factors. However, mesotrione caused severe injury at both sites, while pendimethalin demonstrated potential for effective weed control followed by pyroxasulfone, fomesafen and metolachlor at Bowling Green fields but they caused more than 45% injury at Lexington fields (Maxwell 2016). Following this, pendimethalin was applied as the pre-emergent herbicide at 1.12 kg a.i./ha in a field experiment with Helena variety. At harvest, there was no observed effect on biomass, fiber, or seed yields, indicating that, despite the initial injury caused by the herbicide, hemp plants were able to recover from the early phytotoxicity and stand loss (Anderson 2018). In a study conducted in Romania, an experimental field study was conducted to test 2 different pre-emergent herbicides on the Zenit variety of hemp. S-metolachlor (1.44 kg ai/ha) and aclonifen (1.8 kg ai/ha) had 48% and 72% efficacy in controlling weeds and increased the seed yields by 179% and 181% respectively with no damage to the hemp (Pintilie et al. 2019).

A greenhouse study evaluated sixteen pre-emergent herbicides on the ‘CFX-2’ hemp cultivar, assessing emergence, plant loss, visible injury, and biomass. Results revealed that acetochlor, ethofumesate and pyroxasulfone significantly reduced hemp emergence. Atrazine, ethofumesate, isoxaflutole, mesotrione, metribuzin, pyroxasulfone and saflufenacil caused notable plant loss. Severe injury (> 80%) was observed with ethofumesate, mesotrione, metribuzin and pyroxasulfone, whereas flumioxazin, imazethapyr-ammonium, pendimethalin, quinclorac-methyl and trifluralin showed minimal injury. Acetochlor, dimenthenamid-p, S-metolachlor and sulfentrazone caused moderate biomass reduction and injury symptoms. Based on greenhouse findings (see Table 3), field experiments were conducted in 2019 and 2020 across multiple North Dakota locations using ‘X-59’ hemp. Safest herbicides from each site of action group were applied at both low (1X) and high (2X) rates. Key assessments included visible injury, plant density, and seed yield. Acetochlor and saflufenacil showed greater than 25% injury at higher rate whereas pendimethalin, pyroxasulfone, quinclorac-methyl and trifluralin had minimal injury (< 15%). All herbicide treatments improved seed yield over the weedy control (74.2%), with quinclorac-methyl producing the lowest yield (79.1%) and imazethapyr-ammonium the highest (100.4%). Considering all parameters, pendimethalin, pyroxasulfone and trifluralin were identified as the most promising pre-emergence herbicides for industrial hemp production (Mettler 2021).

Researchers studied hemp’s tolerance to 14 pre-emergence herbicides in greenhouse and field trials. In the greenhouse, chlorimuron, diuron, and pendimethalin showed minimal effects on hemp stand counts, while others (norflurazon, fomesafen, sulfentrazone, pyroxasulfone, clomazone, flumioxazin) reduced stand counts by 43–71%, leaving only 2–4 plants. These treatments also caused significant biomass reductions (33–96%), with pendimethalin, sulfentrazone, diuron, linuron, and flumioxazin causing the least reduction (33–65%). In the field, S-metolachlor and fomesafen resulted in minimal injury (0–15%) and stand counts similar to the untreated check but fomesafen resulted in 55% injury in 2017. Pendimethalin caused a 68% reduction in stand counts, but no significant grain yield differences (97%−106% of non-treated yield)were observed across all herbicides. Overall, S-metolachlor was identified as the safest treatments, with minimal injury and no yield reduction (Byrd 2019; Flessner et al. 2020). A dose–response study showed that biomass reduction exceeded 50% for 23 pre-emergent herbicides applied at the suggested label rate tested on X-59 and in 22 herbicides for CRS-1 hemp varieties. Clopyralid caused ~ 30% reduction in CRS-1 biomass. When applied at a 0.125 × rate, all herbicides caused a biomass reduction of more than 25%, except for saflufenacil in both varieties. These findings highlight the importance of both herbicide dosage and variety dependent tolerance of different herbicides (Ortmeier-Clarke et al. 2022).

A study in Poland on hemp cultivation using the Secuieni Jubileu variety, a Romanian monoecious type, examined three sowing treatments: 20 kg ha^−1^ with herbicide, 30 kg ha^−1^ with herbicide, and 30 kg ha^−1^ without herbicide. In the herbicide-treated plots, Boxer 800 EC (prosulfocarb) was applied at 3–4 L ha^-1^ three days post-sowing to control both dicotyledonous and monocotyledonous weeds. The results demonstrated that higher seeding densities and the application of herbicide significantly reduced weed infestation. The highest seed fraction (2.5 mm) was found in the 30 kg ha^−1^ with herbicide treatment, indicating improved seed quality. However, the combination of herbicide and a seeding density of 20 kg ha^−1^ resulted in the highest seed yield, emphasizing the importance of optimizing seeding density and herbicide use for efficient seed production (Ambroziak et al. 2023). Another research assessed the tolerance of same variety Secuieni—Jubileu to eight pre-emergence herbicides for controlling dicotyledonous and monocotyledonous weeds in Romania. The study found that all eight pre-emergence herbicides caused some level of phytotoxicity in hemp, ranging from 4.67% (oxifluorfen) to 43.00% (metribuzin) at the initial evaluation. Aclonifen was the only herbicide that caused minimal injury (1.67%). At 8 weeks post-treatment, the highest phytotoxicity was observed with metribuzin (35.00%) and clomazone (80.67%). Other herbicides like S-metolachlor (25.33%) and bifenox (11%) caused moderate injury. Lenacil, aclonifen, oxifluorfen and pendimethalin caused less than 10% injury after 8 weeks. Hemp plant height was adversely affected by metribuzin (63.33%) and clomazone (75.33%). Regarding seed production, pre-emergence herbicides like aclonifen (989.0 kg/ha), oxifluorfen (949.7 kg/ha), and S-metolachlor (882.7 kg/ha) yielded higher than the untreated control (766.3 kg/ha). Maximun dicotyledonous weeds were managed by aclonifen (80.0%) and oxifluorfen (84.0%), despite causing minor phytotoxicity in hemp. The study suggested that pre-emergence herbicides like aclonifen and oxifluorfen could be effective for controlling weeds while minimizing hemp injury (Puiu et al. 2023).

A field experiment conducted in Athens, Greece evaluated the effects of the pre-emergence herbicide pendimethalin (applied at 1.6 kg ai ha^-1^) on two monoecious hemp cultivars, Fedora 17 and Uso 31, both widely grown in European climates in 2 growing seasons. Results showed significant differences in the competitive ability and growth response of the cultivars. Fedora 17 demonstrated superior weed suppression compared to Uso 31. The herbicide reduced biomass of perennial and annual weeds by 13% and 38%, respectively, but negatively impacted the growth and yield of Uso 31, with reduced plant height and biomass (27% and 49% in 2019, and 14% and 38% in 2020, respectively). In contrast, while herbicide application in Fedora 17 plots improved plant growth and yield compared to weedy plots, these values were still lower than those achieved through hand hoeing. The herbicide’s negative effects on hemp growth, particularly in Uso 31, may be attributed to phytotoxicity. Yield components further highlighted cultivar-specific responses, with pendimethalin reducing Uso 31 yield but enhancing the productivity of Fedora 17 compared to weedy plots, emphasizing the differential impact of herbicide use on dual-purpose hemp cultivars under field conditions (Kousta et al. 2023). Another study conducted in Greece examined the response of the monoecious industrial hemp cultivar Futura 75 to pre-emergence herbicides and their weed control efficacy. Field experiments tested pendimethalin (1137.5 g ai ha^-1^), S-metolachlor (960 g ai ha^-1^), and aclonifen (1800 g ai ha^-1^). In 2023, these pre herbicides were followed by a post-emergence application of cycloxydim (200 g ai ha^-1^) to address *Sorghum halepense* infestation. In 2022, all pre herbicides caused some degree of hemp growth retardation. S-metolachlor and pendimethalin slightly reduced stand counts, while aclonifen caused leaf yellowing in one experiment. However, in 2023, there was no decreases in plant height or crop establishment, and aclonifen-induced leaf discoloration was less pronounced. Cycloxydim demonstrated excellent control of *S. halepense* without adversely affecting hemp. Despite the initial herbicide injury, hemp plants recovered, achieving higher biomass in both years and increased seed yield in the 2023. This study suggests that aclonifen, S-metolachlor and pendimethalin are viable pre-emergence herbicide options for industrial hemp cultivation, provided precautions are taken on wet and light soils to minimize potential phytotoxicity (Gitsopoulos et al. 2024).

Field studies conducted in 2021 and 2022 assessed the herbicide tolerance of two hemp cultivars, Jinma and Yuma, focusing on various soil-residual herbicides. S-metolachlor, ethalfluralin and a mixture of sulfentrazone with S-metolachlor provided effective weed control (60% to 100%) while showing minimal impact on hemp growth, including germination, plant height, and biomass. Notably, these herbicides showed no significant difference in hemp stand count across cultivars. In contrast, treatments like mesotrione, metribuzin + S-metolachlor and bicyclopyrone + S-metolachlor caused severe toxicity, resulting in zero stand counts and significantly reduced biomass, particularly with mesotrione (19% less than control). The highest biomass yields were observed with S-metolachlor and ethalfluralin treatments, though no significant differences in biomass were found among several other herbicide treatments. Weed control was notably better with the sulfentrazone + S-metolachlor mixture, S-metolachlor, and ethalfluralin, but combinations like bicyclopyrone + S-metolachlor also showed high weed control at 30 DAP, despite toxicity. These results highlight that ethalfluralin, S-metolachlor, and prometryn are viable options for hemp production, while some combinations, like bicyclopyrone + S-metolachlor, should be avoided due to their toxicity. Further research is needed to evaluate herbicide efficacy under varying environmental conditions, ensuring compliance with legal THC content limits (Singh et al. 2024).

A study, conducted in Turkey, evaluated the phytotoxicity of pre-emergent herbicides on the Finola hemp variety, focusing on stem length and diameter, plant height, dry stem, biomass, and fiber yields. Four pre-emergent herbicides—pendimethalin, clomazone, dimethenamid-P and pyroxasulfone were tested in pot and field trials. Pre-emergent applications were made immediately after planting. In the pot experiment, clomazone caused the lowest plant height (66.7% of control) and stem diameter (64%), while pendimethalin led to the shortest root length (63.3%). In field trials, clomazone-treated plots exhibited the lowest technical stem length (53.5%), stem diameter (63.8%), biomass yield (34%), and fiber yield (46.5%). Phytotoxicity symptoms were observed with only clomazone in both pot (70.33%) and field (20.54%) trials. Despite no phytotoxicity in other herbicides, they produced biomass (57.8–95%) and fiber yields (72–98%) that were lower than the weed-free control but higher than the weedy control (biomass: 33.25%, fiber: 44.8%). The study highlights that weed-free conditions remain the most effective for maximizing Finola hemp yield but certain herbicides can be used to improve yields than weedy fields (Kale et al. 2024).

**Fig. 4:**
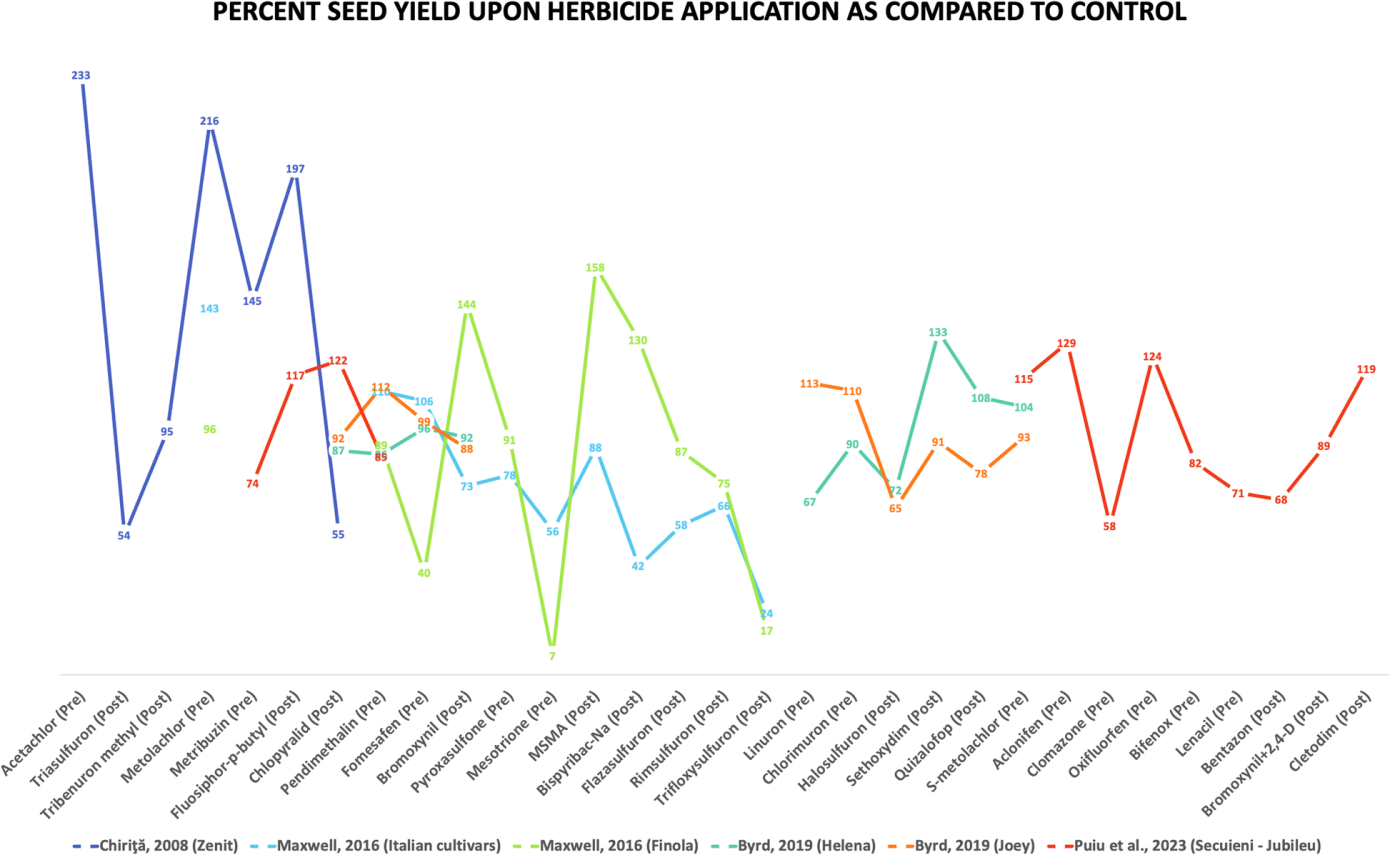
Percent seed yield as compared to control after herbicide application in different studies across various studies and hemp varieties

From different studies, it was observed that hemp’s response to various herbicide applications was highly variable depending on variety selection, application timing and environmental conditions. Overall, pre-emergent pendimethalin application was found to be safe across multiple studies and final seed yield was found to be close to control as shown in Fig. 4. Although, it did cause greater injury in Finola (Maxwell 2016) and Joey (Byrd 2019) but it caused no phytotoxicity with Finola in another study (Kale et al. 2024). Certain herbicides such as mesotrione and metribuzin should be avoided as they cause severe injuries and seed yield loss. Several other herbicides were found to be safe for hemp cultivation across different studies but more research is needed to deem them safe across different hemp fields. Thus, the development of variety-specific recommendations and regional testing programs will be crucial for advancing chemical weed management in hemp production.

### Post-emergent herbicidal control

In 1977, glasshouse and field experiments were initially conducted to investigate the use of post-emergent herbicides for eradicating illicit Cannabis stands. A study in Israel during the same year revealed that most herbicides tested in glasshouse conditions caused significant injury or death to Cannabis plants. Among the herbicides tested, nitrofen was the most well-tolerated and was subsequently recommended for use in legally cultivated Cannabis crops as shown in Table 3 (Horowitz 1977). In 1980, the post-emergent herbicide paraquat was found to be highly effective at killing Cannabis plants. Even at sub-lethal rates, paraquat significantly impacted the plants'biochemical and morphological responses (Coffman & Gentner 1980).

In Romanian field trials with the Zenit variety of hemp, chlopyralid, triasulfuron and tribenuron-methyl resulted in significant injuries to the plant whereas fluosiphor-p-butyl was the safest. Additionally, they also led to decreased seed yields (54.2%−94.7%) as compared to control and 35–52% weed control whereas fluosiphor-p-butyl had 196.9% yield and 39% weed control (Chiriţă 2008). In another study conducted in Romania, an experimental field was used to test 10 different post-emergent herbicides on the Zenit variety of hemp. Several herbicides, including S-metolchlor + piridat, aclonifen, and a combination of S-metolachlor, clopyralid, aminopiralid and picloram, induced various phytotoxicity symptoms. However, the plants recovered within 30 days. The yields from herbicide-treated crops were significantly higher, ranging from 622 kg/ha to 1225 kg/ha, compared to the non-herbicide control, which yielded 558 kg/ha. They showed efficacy levels between 62 and 92%. The combination of S-metolaclor and clopyralid had the highest yield with no damage to hemp and efficacy of 65% (Pintilie et al. 2019). Another research assessed the tolerance of industrial hemp to post-emergence herbicides for controlling dicotyledonous and monocotyledonous weeds in Romania, using the monoecious hemp variety Secuieni—Jubileu. When the hemp plants reached the 8–10 leaf pair stage, 30 days after seeding, five post-emergence herbicides were sprayed. Hemp showed initial phytotoxicity ranging from 8.67% (fluazifop-P-butil to 34.0% (bentazon). Bentazon, on the other hand, caused significant damage (65.0%) after 8 weeks, while fluazifop-P-butil, clopyralid and cletodim showed a lesser impact (< 1.5%) and bromoxinil + acid 2,4-D caused 14.67% phytotoxicity. Among these, clopyralid (936.7 kg/ha), cletodim (911.7 kg/ha) and fluazifop-P-butil (893 kg/ha) were the most seed yielding, with the untreated check yielding 741.7 kg/ha. Monocotyledonous weeds were effectively controlled by cletodim (89.67%) and fluazifop-P-butil (91.33%), while dicotyledonous weeds were managed by clopyralid (61.0%) with minimal injury to hemp. The study proposed a weed management strategy for hemp cultivation, recommending the use of aclonifen or oxifluorfen as pre-emergence herbicides, followed by selective post-emergence applications of clopyralid for dicotyledonous weeds and cletodim or fluazifop-P-butil for monocotyledonous weeds (Puiu et al. 2023).

In USA, Maxwell conducted a study testing 6 post-emergent herbicides at two different locations (Bowling Green and Lexington). The results showed significant variation between the locations due to these factors. However, trifloxysulfuron, flazasulfuron and rimsulfuron caused significant injury at both sites, while MSMA and bromoxynil demonstrated potential for effective weed control. Bispyribac-Na exhibited varying injury at two locations (Maxwell 2016). Flessner et al. studied hemp’s tolerance to 14 post-emergence herbicides in greenhouse and field trials. Quizalofop, sethoxydim, acifluorfen, fomesafen, clopyralid, and halosulfuron had no effect on biomass, while bentazon, pyrithiobac, bromoxynil, thifensulfuron, imazaquin, linuron, imazethapyr, and chlorimuron reduced biomass by 40–56%. In field trials, quizalofop, clopyralid, sethoxydim, and bromoxynil caused < 20% injury at all evaluation times and had no significant impact on yield, while halosulfuron caused severe injury (60%) and reduced yield. Overall, quizalofop, clopyralid, bromoxynil, and sethoxydim were identified as the safest treatments, with minimal injury and no yield reduction (Byrd 2019; Flessner et al. 2020). A study examining the impact of various environmental stresses found that a post-emergent application of glyphosate led to a reduction in cannabinoid production, along with necrosis and browning of high cannabidiol hemp plants by the second week (Toth et al. 2021). Research on pesticide metabolism showed that the herbicide flurtamone significantly affected the growth of hemp plants. The first pair of leaves, which had already unfurled prior to incubation, dried out rather than getting bleached due to the declining overall health of the plant. However, flurtamone underwent degradation through the activity of monooxygenase enzymes (Hillebrands et al. 2021).

In a greenhouse study, hemp ('Anka') seeds were treated with mesotrione, glufosinate, clopyralid, fomesafen, dicamba, rimsulfuron, bromoxynil, glyphosate, bentazon, prometryn, and carfentrazone at the 4- to 8-leaf stage, along with a non-treated check for comparison. Herbicides were applied at label-recommended rates, with irrigation withheld for 24 h post-application to ensure foliar absorption. Injury increased over time in most treatments. Bromoxynil caused the least injury (13%, with minor stunting and chlorosis) and maintained 85% of the untreated biomass, while clopyralid caused 44% injury (elongated stems, leaf rolling) and retained 75% biomass. All other herbicides caused 85%−100% injury, with effects such as bleaching, chlorosis, necrosis, and severe deformations, and biomass retention did not exceed 30%. These findings suggest potential hemp tolerance to bromoxynil and clopyralid but significant susceptibility to other tested herbicides (Sosnoskie & Maloney 2021).

For post-emergence herbicide testing, a greenhouse study assessed 26 herbicides with different modes of action on the ‘CFX-2’ hemp cultivar. Bromoxynil and clopyralid caused minimal injury, while herbicides such as fluroxypyr-methyl, fomesafen-sodium, glufosinate-ammonium, glyphosate-potassium, halauxifen-methyl, paraquat, tembotrione and topramezone exhibited greater than 75% biomass reduction correlated with injury. Based on greenhouse findings (Table 3), field experiments were conducted in 2019 and 2020 across multiple North Dakota locations using ‘X-59’ hemp. At 21 days after treatment, herbicides caused visible injury, with atrazine, cloransulam-methyl and oxyfluorfen being the most damaging whereas clopyralid caused minimal injury. By 35 th day, cloransulam-methyl and oxyfluorfen significantly reduced plant density. Seed yield was adversely affected by cloransulam-methyl (27.3% of weed free control) and oxyfluorfen (45.1%), but showed good yield with quinclorac-methyl (73.5%), imazamox-ammonium (75.6%), clopyralid (80.1%), atrazine (84%) and bromoxynil (95.3%) which was still greater than non-treated weedy fields (68%). Field trials confirmed that herbicides like clopyralid and bromoxynil were the least injurious, showing promising tolerance in hemp (Mettler 2021).

A dose–response study showed that biomass reduction exceeded 75% for 19 post-emergent herbicides applied at the suggested label rate tested on both the X-59 and CRS-1 *Cannabis sativa* varieties. However, clopyralid and clethodim showed less than 25% biomass reduction. When applied at a 0.125 × rate, all herbicides caused a biomass reduction of more than 25%, except for clopyralid and clethodim. These findings suggest that post-emergent herbicides causes more injury to the hemp plants (Ortmeier-Clarke et al. 2022).

Another research explored the effects of five non-selective postemergence bioherbicides (D-limonene, ammonium nonanoate, pelargonic acid, caprylic + capric acid, and acetic acid) and two mulches (rice hulls at depths of 1.3 cm and 2.5 cm, and mini-pine bark nuggets) on the growth, floral biomass, and cannabinoid content of the hemp variety ‘Wife.’ Sixty vegetatively propagated hemp plants were cultivated in 4 L containers under greenhouse conditions, and treatments were applied three weeks after transplanting during flower initiation. Rice hull mulch at both depths resulted in slightly reduced plant growth indices compared to the control. Lemon grass oil and caprylic + capric acid caused the highest crop injury, at 14.1% and 12.6%, respectively, while acetic acid and ammonium nonanoate caused lower injury levels (7.9% and 5.9%). Despite these variations, all herbicide treatments exhibited plant growth indices similar to the control, with no significant variations detected in THC, CBD, CBG, or CBC content. Although weed control efficacy was not evaluated, the study suggests that these herbicides pose minimal risk to cannabinoid synthesis in container-grown hemp and provides a foundation for future weed management research (Petrusha et al. 2023).

In a series of greenhouse and growth chamber studies, the response of hemp variety ‘Tangerine’ to herbicide exposure was examined, focusing on 2,4-D and dicamba at varying off-target rates (from 1 × to 1/100,000 × labeled rate). The objectives were to evaluate herbicide effects on injury, plant height, branching, and reproductive parameters, with applications occurring during the early vegetative stage. Results showed that at the 1 × rate, 2,4-D and dicamba caused significant injury—68% and 78%, respectively—28 days after treatment. Height reductions at trial termination were 19 cm for 2,4-D, 25 cm for dicamba, and 9 cm for the 1/10 × dicamba rate. However, no significant effects on branching or plant weight were observed. The growth chamber study revealed that 2,4-D at the 1 × rate caused 82% injury, but no significant differences in height, fresh weight, or CBD levels compared to the control. These findings suggest that low off-target rates of 2,4-D and dicamba may cause visible injury but have limited long-term effects on hemp’s growth and CBD production, especially when exposure occurs early in the vegetative stage. However, further research on different hemp varieties and field conditions is needed to understand the full impact of these herbicides, particularly at later growth stages (Essman 2022; Essman et al. 2024).

A controlled study conducted in 2022 and 2023 assessed the tolerance of two industrial hemp varieties, NWG2730 (cultivar A) and NWG452 (cultivar B), to multiple herbicides, including fluazifop, clethodim, fenoxaprop, quizalofop, pinoxaden, fluazifop + fenoxaprop, and sethoxydim. These herbicides were applied at four different rates (0.5-, 1-, 2-, and 4-times the highest labeled rate) when the plants reached a height of 20–25 cm (two to three pairs of true leaves). Among the treatments, clethodim and pinoxaden induced the most pronounced visual injury and biomass reductions, with estimated 5% biomass reduction rates of approximately 131 and 192 g ai ha^-1^ for clethodim, and 60 and 39 g ai ha^-1^ for pinoxaden in cultivars A and B, respectively. Meanwhile, quizalofop, fluazifop, and sethoxydim exhibited minimal negative effects, indicating their potential as ACCase-inhibiting herbicides for hemp production. The findings underscored differences in herbicide tolerance between cultivars and active ingredients (Zaric 2023). Additionally, the study examined hemp’s susceptibility to herbicide drift from products commonly used in corn and soybean fields, including lactofen, glyphosate, dicamba, mesotrione, 2,4-D, imazethapyr, and glufosinate. Drift simulations were performed in a wind tunnel on hemp plants at the 20–25 cm growth stage, and biomass reductions were evaluated 21 days post-application. Nozzle design played a crucial role in drift dispersion, with TP nozzles allowing 5% of spray deposits to reach 5.9 m downwind, compared to 2.0 m for AI nozzles. Glyphosate, glufosinate, and mesotrione caused the greatest biomass reductions, with 50% reductions occurring at 19.3, 8.7, and 9.3 m downwind using TP nozzles, and at 4.1, 4.0, and 2.9 m downwind with AI nozzles. These results emphasize the potential risk of herbicide drift impacting nearby hemp fields when applied at airspeeds of ≥ 3.6 m s^-1^ (Zaric 2023; Zaric et al. 2024).

Researchers studied the application of various treatments before and after transplanting on floral hemp seedlings (‘Von’ and ‘Sunbelt Crush’). Results indicated that isoxaflutole, isoxaben, and halosulfuron-methyl were unsuitable for pretransplant applications in hemp cultivated for flower production. Some herbicides like acetochlor, pendimethalin, and flumioxazin showed potential for further investigation, while others required significantly lower application rates. In posttransplant trials, herbicides such as clethodim, pendimethalin, and S-metolachlor caused minimal injury, under 5%, with no significant impact on height or flower yield. However, halosulfuron, metribuzin, imazethapyr, prometryn, and trifloxysulfuron caused severe injury (over 50%), reducing height and biomass. Despite this, imazethapyr yielded results within 50% of the control in dry flower production. Based on these findings, further research is needed to assess the viability of acetochlor, pendimethalin, and clethodim for floral hemp production (Wright 2022; Wright-Smith et al. 2024).

Studies in Russia demonstrated that clopyralid (Lontrel Grand) and hizalophop-P-ethyl (Miura) effectively controlled weeds and increased hemp biomass, leaf surface area, seed and stem yields (Pluzhnikova et al. 2023a, 2023b; Pluzhnikova & Kriushin 2024). Research conducted in Turkey, evaluated the phytotoxic effects of post-emergent herbicides on the Finola hemp variety. Four post-emergent herbicides—quizalofop-P-ethyl, bentazone, halosulfuron-methyl, and bromoxynil were tested in pot and field trials where applications were conducted 30 days later. In the pot experiment, bentazone caused the lowest plant height (54.8% of control) and stem diameter (60.3%), while bromoxynil led to the shortest root length (70.53%). In field trials, bromoxynil-treated plots exhibited the lowest technical stem length (34.61%), stem diameter (57.87%), biomass yield (23.8%), and dry stem yield (35.93%), while bentazone-treated plots produced the lowest fiber yield (30.21%). Phytotoxicity symptoms were observed with only bromoxynil and bentazone in both pot (58.66% and 80.25%) and field (58.44% and 75.52%) trials. Despite no phytotoxicity in other herbicides, they produced biomass and fiber yields that were lower than the weed-free control but higher than the weedy control. This study also highlights that maintaining weed free fields is crucial for maximizing benefits from hemp production (Kale et al. 2024).

Across different studies, grass specific herbicides such as quizalofop, clethodim, sethoxydim and fluazifop provided the safest and effective control of weed species causing minimal harm to the hemp plants owing to different enzyme chemistries. For broadleaf control, most post-emergent herbicides caused greater injury than pre-emergent herbicides, potentially due to direct contact between the herbicide molecules and plant tissue causing higher herbicidal intake by the plant. Clopyralid and bromoxynil was found to be relatively safe for broadleaf control among the tested herbicides and good seed yields in different trials as shown in Fig. 4. In contrast, trifloxysulfuron, metribuzin, glyphosate, imazethapyr and halosulfuron were found to cause moderate to severe injury in multiple trials and caused seed yield loss as shown in Fig. 4. Further testing is required for these herbicides across different regions and varieties to be considered safe for mass usage.

## Future perspectives

Several areas of research and development hold promise for advancing weed management in hemp cultivation. 1. Systematic testing of herbicides on different hemp varieties is crucial to expand the range of approved chemical options. Research should focus on identifying herbicides with minimal phytotoxicity and maximum efficacy across various environmental conditions and weed pressures. Collaborative efforts between regulatory agencies, researchers, and industry stakeholders can accelerate the approval process for safe and effective herbicides. 2. Further refinement of planting densities, row spacing, and crop rotation schedules can enhance hemp’s natural weed-suppressive abilities while maintaining yield and quality. 3. Understanding weed species composition and dynamics in hemp fields under varying climates and cultivation systems is essential. Comprehensive studies on weed species composition, weed herbicide resistance and competitive dynamics across diverse climates and cultivation systems are needed to inform region-specific management strategies. 4. Breeding programs should prioritize the development of hemp cultivars with enhanced weed-suppressive traits, such as faster early growth, higher biomass production, and allelopathic properties and herbicide tolerant varieties. By addressing these priorities, the hemp industry can develop robust weed management systems that maximize productivity and profitability while supporting environmental sustainability. Through continued research and innovation, hemp can solidify its position as a cornerstone crop for future agricultural systems.

## Conclusion

This review highlights the significant challenges and opportunities associated with weed management in industrial hemp cultivation. Hemp’s rapid growth, dense canopy, and adaptability make it an inherently competitive crop against weeds, offering the potential to reduce reliance on chemical herbicides. However, effective weed control remains crucial, particularly during the crop’s early growth stages when weeds can severely impact germination, plant density, and yield. Cultural practices, such as optimized planting densities, row spacing, and crop rotation, have proven effective in leveraging hemp’s natural weed-suppressive capabilities. Mechanical methods like tillage and hand weeding offer additional solutions but are often labor-intensive and less feasible for large-scale operations. Chemical control, while widely used in conventional agriculture, remains underdeveloped in hemp due to the limited availability of approved herbicides and the crop’s sensitivity to many chemical inputs. Despite some promising results with pre- and post-emergent herbicides like pendimethalin, quizalofop, fluazifop, bromoxynil and clopyralid, further research is needed to address regional weed pressures, environmental variability, and variety-specific tolerances. Integrated Weed Management strategies that combine cultural, mechanical, and chemical approaches provide the most promising path forward. These systems balance the need for effective weed suppression with the goal of minimizing environmental impacts, aligning with hemp’s role as a sustainable crop. Importantly, hemp’s natural weed suppression through biomass production and canopy closure underscores its potential to support sustainable farming practices while reducing the dependency on chemical herbicides.

## Data Availability

No datasets were generated or analysed during the current study.
